# B-cell abundance in perivascular cuffs associates with local lesion activity in multiple sclerosis

**DOI:** 10.1186/s40478-025-02208-4

**Published:** 2026-02-05

**Authors:** Hendrik J. Engelenburg, Esmée Westenbrink, Eline Runderkamp, Ana M. Marques, Marvin M. van Luijn, Cheng-Chih Hsiao, Inge Huitinga, Jörg Hamann, Joost Smolders

**Affiliations:** 1 Neuroimmunology Research Group, Netherlands Institute for Neuroscience, Meibergdreef 47, 1105 BA Amsterdam, The Netherlands; 2 Department of Immunology, MS Center ErasMS, Erasmus MC, University Medical Center Rotterdam, Rotterdam, The Netherlands; 3 Swammerdam Institute for Life Sciences, University of Amsterdam, Amsterdam, The Netherlands; 4 Department of Experimental Immunology, Amsterdam Institute for Immunity and Infectious Diseases, Amsterdam University Medical Centers, Amsterdam, The Netherlands; 5 Department of Neurology, MS Center ErasMS, Erasmus MC, University Medical Center Rotterdam, Rotterdam, The Netherlands

**Keywords:** Perivascular cuffs, Multiple sclerosis, B cells, T cells, EBV, BTK

## Abstract

Perivascular aggregations of leukocytes, referred to as perivascular cuffs, are a pathological phenomenon in progressive multiple sclerosis (MS). Perivascular cuffing is an exaggerated form of compartmentalized inflammation present in progressive disease. By studying traits of perivascular cuffs, this study aims to elucidate processes within the perivascular niche of the MS brain. We characterized n = 255 MS donors from the Netherlands Brain Bank for the presence of perivascular cuffs and investigated their association with clinical and pathological donor characteristics. Furthermore, we examined the proportional abundance of different cell types and functional markers in n = 457 perivascular cuffs present in different lesion stages within a cohort of n = 18 MS brain donors. MS donors with detected perivascular cuffs (25.5%) showed a higher brainstem lesion count. Within these cuffs, there was a relative increase of CD4^+^ compared to CD8^+^ T cells, and a higher B-cell abundance in active and mixed active/inactive MS lesions specifically. All perivascular cuffs were inflammatory active (TNF, PCNA, HLA) and contained antibody-secreting cells, which correlated with the presence of CD4^+^ T cells. In a different cohort, CNS-isolated viable B cells tested positive for EBV with qPCR (6/6 MS donors). In these donors, EBV/LMP-1^+^ B cells were detected in the meninges. In the cuffing cohort, LMP-1^+^ B cells were only present sparsely (13/122 perivascular cuffs of 6/11 donors). Lastly, Bruton’s tyrosine kinase (BTK) was abundant in B cells and macrophages within all perivascular cuffs. Our comprehensive characterization of perivascular cuffs in advanced MS links them with disease severity and supports a local interaction between CD4^+^ T and B cells to be associated with lesion presence. Our data indicate that this can happen irrespective of local EBV presence, and supports the targetability of perivascular cuffs by brain-penetrating BTK inhibitors.

**Supplementary Information:**

The online version contains supplementary material available at 10.1186/s40478-025-02208-4.

## Introduction

Multiple sclerosis (MS) is an inflammatory disease of the central nervous system (CNS). Clinically, MS is characterized by accumulation of disability in tandem with inflammatory attacks resulting in neurological deficits [1]. Genetic variants associated with MS susceptibility underline the importance of lymphocytes in disease etiology [2], whereas epidemiological studies show a major impact of the B-cell lymphotropic Epstein-Barr virus (EBV) as virtual prerequisite for developing MS [3].

In non-MS human brain white matter (WM), perivascular CD8^+^ and to a lesser extent CD4^+^ tissue-resident memory T (T_RM_) cells are prevalent along with low numbers of B cells, proposing that the perivascular space (PVS) between endothelial cells and glia limitans serves as niche for adaptive immune surveillance of the brain [4]. In MS, the number of perivascular T_RM_ cells is increased compared to control brain donors [5]. In lesions, these cells not only cluster around blood vessels but also infiltrate the parenchyma [5, 6]. Likewise, increased numbers of PVS B cells can be found in MS, albeit that they remain less abundant compared to T cells [5, 7, 8]. These B cells mostly adopt a CD38^high^ antibody-secreting cell phenotype, synthesize immunoglobulin G (IgG), and correlate, in MS lesions, with the relative abundance of CD4^+^ T cells [8, 9]. These findings put forward the PVS as a possible hotspot of T–B-cell interactions in MS [10].

Exaggerated accumulation of lymphocytes in the PVS, also known as perivascular cuffs, is a sporadic MS characteristic of which the relevance of cellular composition and interaction for the local disease process of MS is poorly understood. It has been reported that perivascular cuffs in demyelinated areas contain Ig-secreting plasma cells and localize mostly in the highly affected supratentorial regions of people with progressive MS [11, 12]. Moreover, we previously found that the presence of perivascular cuffs is associated with a more severe disease progression [13]. Whether the cellular composition and activity of perivascular cuffs differs from that in PVS without cellular aggregations is not yet known. Initial work showed that within perivascular cuffs there is a presence of Ki-67^+^ proliferating cells [5]. Moreover, T cells in perivascular cuffs frequently express E-cadherin and its ligand CD103, which may contribute to their local retention [14]. Using the MS cohort of the Netherlands Brain Bank (NBB), we here set out to test whether the presence and composition of perivascular cuffs associates with MS WM lesion presence and activity and to explore the different cell types and their spatial association with different lesion types.

## Methods

### Donor and sample characteristics

Informed consent was given by the donors for brain autopsy and for the use of material and clinical data for research purposes in compliance with national guidelines. The NBB autopsy procedures were approved by the Medical Ethics Committee of the VU Medical Center, Amsterdam, The Netherlands. The donors came to autopsy between 1990 and 2022.

MS brain donors from the NBB (n = 255) were included in the analysis of pathological and clinical characteristics. Clinical files were collected retrospectively by the NBB, and diagnosis of MS was confirmed by a certified neuropathologist. Pathological characteristics, such as the presence of perivascular cuffs, were acquired as described previously [5, 15, 16]. For instance, the microglia/macrophage activation score (MMAS) is the average semi-quantitative score of microglia/macrophages in active and mixed active/inactive lesions, where thin and ramified microglia/macrophages are counted as 0, ameboid microglia/macrophages with few ramifications are counted as 0.5, and foamy microglia/macrophages are given a score of 1. For the immunohistochemistry, 21 subcortical WM tissue blocks containing perivascular cuffs of n = 18 MS donors from the NBB were analyzed (Supplementary Table 1, Supplementary Material 1). These blocks were selected based on availability of tissue within our cohort and to include as many lesion types as possible. 457 perivascular cuffs sampled from normal-appearing WM (NAWM; n = 17 donors), reactive sites (n = 4 donors), active lesions (n = 8 donors), mixed active/inactive lesions (n = 8 donors, 58,8% was sampled from the lesion core, 41,2% from lesion rims or the direct perilesional area), inactive lesions (n = 4 donors), and remyelinated lesions (n = 8 donors) were studied [16].

### Immunohistochemistry

For immunohistochemistry, tissue was cut from paraffin-embedded (8-µm) tissue blocks. Perivascular cuff presence was assessed based on CD3 or HLA/PLP immunohistochemistry with a hematoxylin counterstain, characterized when the number of cells could span ≥ 2 layers around a blood vessel. For pathological characterization, a donor was annotated as positive if any sampled block contained a perivascular cuff.

Adjacent tissue sections were stained for the presence of T cells, B cells, NK cells, perivascular macrophages, EBV (LMP-1, EBNA2), and functional markers (PCNA, IgG, TNF, BTK). For LMP-1 staining, a blocking step with 3% H_2_O_2_ in methanol was performed for 30 min at room temperature. Antigen retrieval was done for 10 min at 700 W, and sections were cooled for 45 min and subsequently incubated with blocking buffer (10% horse serum, 1% BSA, and 0.5% Triton X-100 in Tris-buffered saline, pH7.6) for 60 min. Primary antibodies were incubated overnight at 4°C (Supplementary Table 2, Supplementary Material 1). Then, blocking with 1% H_2_O_2_ was performed. Sections were incubated with a biotinylated secondary antibody for 60 min (1:400) followed by incubation for 45 min with avidin-biotin horseradish peroxidase complex (Elite ABC-HRP kit; Vector Laboratories). IgG staining was amplified with 0.01% biotinylated tyramide in 0.001% H_2_O_2_ borate buffer for 10 min followed by another ABC-HRP incubation (45 min). All stainings were visualized with 3’3-diaminobenzidine and counterstained with hematoxylin. Positive and negative controls of EBV stainings are shown in Supplementary Fig. 1a (Supplementary Material 1). Brightfield images were acquired using the AxioScan Z1 microscope (Zeiss) at 20× magnification.

### Immunofluorescence

For immunofluorescence, sections were incubated with a compatible fluorophore or a compatible biotinylated secondary antibody followed by a streptavidin-conjugated fluorophore for 60 min (Supplementary Table 2, Supplementary Material 1). In EBV double stainings, a directly conjugated CD79a-antibody with Alexa Fluor 647 was used. IgG staining was enhanced using tyramide signal amplification as described above and visualized using a streptavidin-conjugated fluorophore (1:800) for 60 min. All fluorescent stainings were incubated with Hoechst (33342; Thermo Fisher Scientific). Fluorescent images were visualized using confocal microscopy on a STEDYCON microscope (Abberior Instruments) at 40× or 63× magnification.

### Image analysis

Images were analyzed using QuPath (v0.5.0) and ImageJ (v1.54p). Cuffs and lesions were manually annotated, and objects were created using cell detection based on the hematoxylin signal. Objects were classified as positive or negative using a single-measurement thresholder using the DAB channel (Supplementary Fig. 1b, Supplementary Material 1). Per-staining settings were determined through visual inspection. NKp46 was not quantified due to low number of positive cells. For IgG, perivascular cuffs were semi-quantitatively characterized as negative, faint-positive, or positive.

### Flow cytometry

CD8^+^/CD4^+^ T-cell ratio from previous analyses was reanalyzed in FlowJo software (BD Biosciences) [5, 17, 18]. Shortly, WM from control and MS cases and macroscopically visible MS lesions was dissected at autopsy and stored at 4°C in Hibernate A medium (Thermo Fisher Scientific). The remaining tissue was enzymatically dissociated, and mononuclear cells were separated from the suspension by Percoll (GE Healthcare) gradient centrifugation. Cells were cryopreserved in liquid nitrogen until use. Single-cell suspensions were blocked with FcR Blocking Reagent (Miltenyi Biotec) and stained with cocktails of fluorochrome-conjugated antibodies, as described in the original research. Stained cells were acquired using the LSRFortessa (BD Biosciences).

### B-cell isolation and EBV qPCR

Tissue was dissected at autopsy and stored in Hibernate A medium until processed within 24 h (Supplementary Table 1, Supplementary Material 1). Cells were isolated from NAWM, WM lesions, CSF, leptomeninges, and peripheral blood as described before [17, 19]. Single cell suspensions were blocked with FcR Blocking Reagent and stained with a cocktail of fluorochrome-conjugated antibodies (Supplementary Table 2, Supplementary Material 1). B cells (CD19^+^, CD20^+^, and CD3^−^CD38^hi^ CD27^+^) were sorted on an Aria II cell sorter (BD Biosciences; Supplementary Fig. 1c, Supplementary Material 1). DNA was extracted using a GenElute™ Mammalian Genomic DNA Miniprep Kit (Sigma-Aldrich) according to manufacturer’s instructions (Supplementary Table 3, Supplementary Material 1). EBV DNA presence was determined as described previously using maximum input DNA [20]. Shortly, DNA was added to a reaction mixture containing primers and probes for *BALF5* and *B2M* supplemented with TaqMan™ Universal Master Mix II, no UNG (Thermo Fisher Scientific). Samples were run on a QuantStudio™ 5 machine (Thermo Fisher Scientific).

### Statistical analysis

Pathological characteristics were analyzed using a Kruskal–Wallis with Dunn’s post hoc test, a Wilcoxon rank sum test (flow cytometry), a chi-squared test (sex, MS type, nodules), or a generalized linear model using a Gaussian (age, gARMSS), quasi-binomial (lesion proportions, MMAS) or quasi-Poisson distribution (lesion load, cortical lesion rate). Data obtained from immunohistochemistry was tested using generalized linear mixed models using a Gaussian (CD8^+^/CD4^+^ T-cell ratio) or negative binomial distribution, with donor as covariate and perivascular cuff size as offset. Negative binomial distribution was chosen based on evaluation of model fit using negative binomial or beta-binomial models and different packages through the ‘performance’ R package (v0.12.3). Visualization of mixed linear models was performed using estimated marginal means, a predicted adjusted mean within a model that takes into account nested data and variance in perivascular cuff size, and a per-donor average of positive-cell ratio. Multiple testing correction was done using the Benjamini–Hochberg false discovery rate. Correlations were tested using a Pearson correlation. For correlations, a CD8^+^/CD4^+^ T-cell balance was used because direct associations with CD4 presence would have a bias to negative correlations. The use of a CD8^+^/CD4^+^ ratio therefore serves as an internal normalization. The criterion was set at α = 0.05. Statistical analysis was performed in GraphPad Prism (v10.5.0) or Rstudio (version 2024.04.0; Posit Software) for R (version 4.3.1), using key packages tidyverse, lme4, emmeans, and ggplot2.

## Results

### Perivascular cuff presence correlates with exaggerated MS WM pathology

**Fig. 1:**
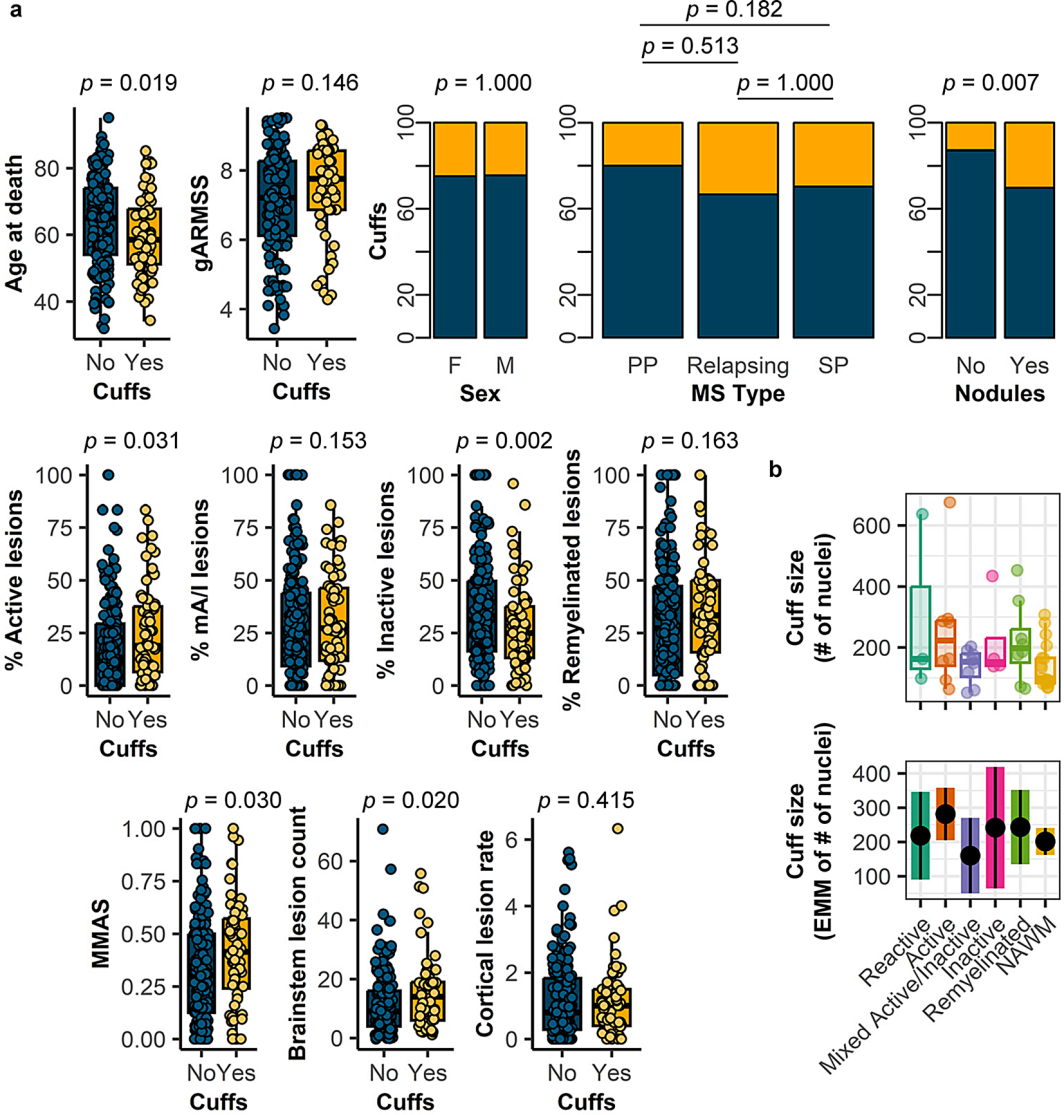
Donors with perivascular cuffs have a more active disease. **a** Clinical and pathological characteristics related to the presence of perivascular cuffs. Dark blue indicates samples without perivascular cuffs, whereas orange represents samples with perivascular cuffs. **b** Perivascular cuff size per WM lesion type compared to NAWM. Plots are visualized as individual data point in a scatter plot, as average ratio of positive cells/counted nuclei per donor and estimated marginal means of absolute positive cell counts, a predicted adjusted mean within a model that takes into account nested data. Box plots show median and interquartile range. Statistics were performed using a chi-squared test (sex, MS type, nodules) or a generalized linear (mixed) model using a Gaussian (age, gARMSS), quasi-binomial (lesion proportions, MMAS), quasi-Poisson (lesion load, cortical lesion rate), or negative binomial distribution (cuff size). EMM, estimated marginal means; F, female; gARMSS, global age-related MS severity score; M, male; mA/I, mixed active/inactive lesions; MMAS, microglia/macrophage activation score; PP, primary progressive; SP, secondary progressive

Within the NBB MS autopsy cohort, we identified perivascular cuffs in tissue blocks from 65/255 (25.5%) donors. Clinical and pathological characteristics of donors are shown in Fig. 1a. Although perivascular cuff presence did not associate with disease severity (gARMSS), sex, or MS type (progressive/relapsing), a 4.3-years lower average age at death was noted in donors with perivascular cuffs (*p* = 0.019). Pathologically, there were no cuff-associated differences in proportion of mixed active/inactive and remyelinated lesions, or in cortical lesion rate. Donors with perivascular cuffs showed an increase in microglia/macrophages with a foamy morphology (MMAS; *p* = 0.030), a higher proportion of active lesions (*p* = 0.031), a decrease in inactive lesions (*p* = 0.002), a higher brainstem lesion count (*p* = 0.020), and a higher abundance of nodules–small microglia clusters associated with lesion initiation (*p* = 0.007) [21]. Perivascular cuffs were characterized in different WM lesion types and in NAWM. No difference in perivascular cuff size, as reflected by number of nuclei, was found between different lesion types and NAWM (Fig. 1b). Overall, MS donors with perivascular cuffs at autopsy displayed an exaggerated WM pathology.

### Perivascular cuffs display a relative increase in CD4^+^ T cells

**Fig. 2:**
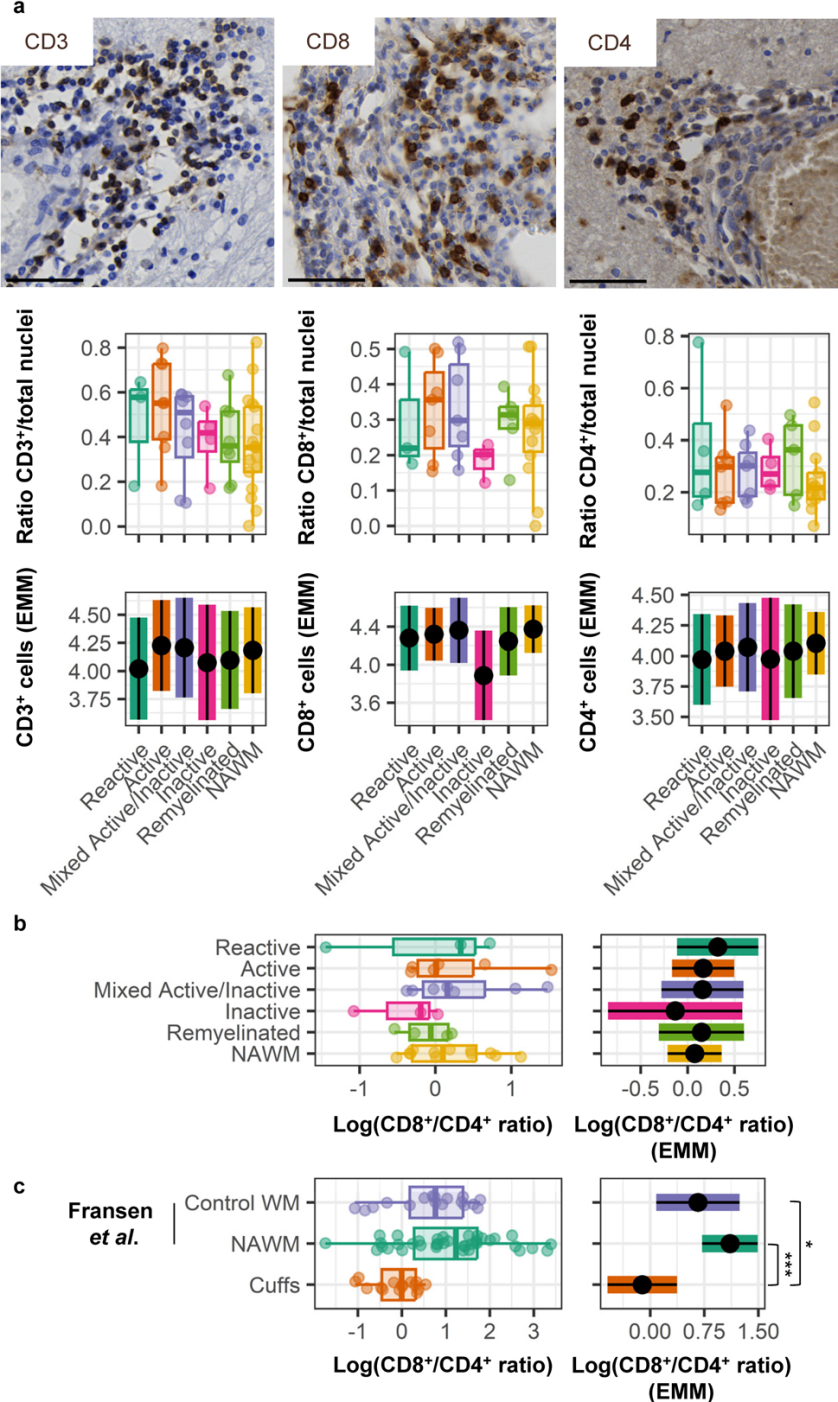
Increased relative abundance of CD4^+^ T cells in perivascular cuffs compared to NAWM. **a** Representative stainings and quantification of CD3^+^, CD8^+^, and CD4^+^ T cells in perivascular cuffs. **b** Log-transformed CD8^+^/CD4^+^ T-cell ratio per WM lesion type compared to NAWM. **c** CD8^+^/CD4^+^ T-cell ratio as detected in perivascular cuffs by immunohistochemistry compared to whole slide quantification in NAWM and control WM from Fransen et al. [5]. Scale bars indicate 50 μm. Plots are visualized as individual data point in a scatter plot, as average ratio of positive cells/counted nuclei per donor and estimated marginal means of absolute positive cell counts, a predicted adjusted mean within a model that takes into account nested data and variance in perivascular cuff size. Box plots show median and interquartile range. Statistics were performed using a linear mixed model using a gaussian (CD8^+^/CD4^+^ ratio) or negative binomial (CD3^+^/CD4^+^/CD8^+^) distribution. Multiple testing correction was performed using a Benjamini-Hochberg FDR. ***p < 0.001; *p < 0.05; EMM, estimated marginal means; NAWM, normal-appearing white matter

To investigate the immunological processes within perivascular cuffs, we assessed their cellular composition. The presence of T cells, both CD4^+^ and CD8^+^, was similar between perivascular cuffs in lesions and NAWM (Fig. 2a). On average, the prevalence of CD8^+^ cells in perivascular cuffs was similar to that of CD4^+^ cells (ratio 1.07; Fig. 2b), which differs from the mean CD8^+^/CD4^+^ T-cell ratio of 1.58 in WM from non-MS donors (*p* = 0.036, Fig. 2c) or the mean CD8^+^/CD4^+^ T-cell ratio of 2.15 previously found in NAWM of the entire MS cohort of the NBB (*p* < *0.*0001; Fig. 2c). To explore whether CD4^+^ T-cell abundance is a trait of donors or is related spatially to perivascular cuffs, we reanalyzed previous datasets [5, 17, 18]. Donors with perivascular cuffs had no altered CD8^+^/CD4^+^ T-cell ratio in NAWM compared to non-cuffing donors (*p* = 0.326) or non-MS control donors (*p* = 0.999) as analyzed with immunohistochemistry (Supplementary Fig. 2a, Supplementary Material 1). Using flow cytometry of NAWM-derived cells, no difference in CD8/CD4 ratio between donors with perivascular cuffs compared to donors without perivascular cuffs (*p* = 0.282) or compared to non-MS controls was observed (*p* = 0.116; Supplementary Fig. 2b, Supplementary Material 1) [5, 17, 18]. Therefore, the relatively higher abundance of perivascular CD4^+^ T cells was interpreted as a spatial trait of cuffs not associated with lesion stage in MS.

### B-cell abundance in perivascular cuffs associates with MS lesions

**Fig. 3:**
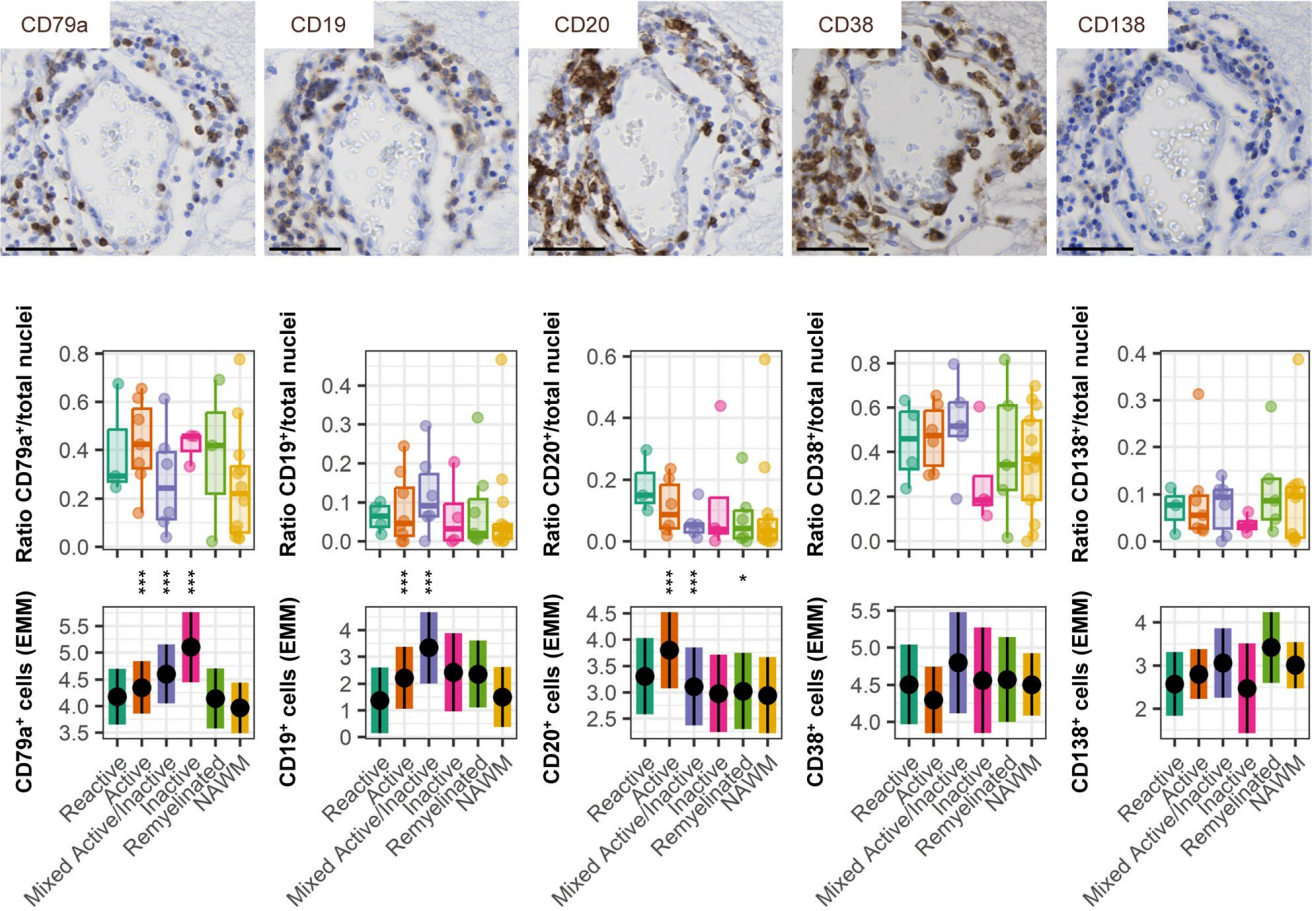
B cells accumulate in perivascular cuffs associated with demyelination. Representative stainings and quantification of CD79a, CD19, CD20, CD38 and CD138 in perivascular cuffs per WM lesion type compared to NAWM. Scale bars indicate 50 μm. Plots are visualized as average ratio of positive cells/counted nuclei per donor and estimated marginal means of absolute positive cell counts, a predicted adjusted mean within a model that takes into account nested data and variance in perivascular cuff size. Box plots show median and interquartile range. Statistics were performed using a linear mixed model using a negative binomial distribution. Multiple testing correction was performed using a Benjamini-Hochberg FDR. ***p < 0.001, *p < 0.05; EMM, estimated marginal means; NAWM, normal-appearing white matter

We next studied the distribution of B-cell lineage populations in perivascular cuffs within lesions compared to NAWM (Fig. 3). Relative numbers of the pan-B cell marker CD79a were high in perivascular cuffs within active (*p* < 0.001), mixed active/inactive (*p* < 0.001), and inactive (*p* < 0.001) lesions. CD19^+^ B cells were also increased in active lesions (*p* = 0.001) and mixed active/inactive (*p* < 0.001) compared to NAWM. In line, the presence of CD20^+^ B cells, expressed by a subpopulation of CD19^+^ B cells, was increased in reactive (*p* < 0.001) and active lesions (*p* < 0.001) compared to NAWM. Although CD20^+^ cell presence was also increased in remyelinated lesions, the effect size was very small (difference in estimated means 0.08; *p* = 0.016). Since CD19 and CD20 are not retained during plasma cell differentiation, we investigated markers associated with B-cell differentiation. CD38^+^ cell presence, which is a memory B cell marker that at high expression-levels is associated with antibody-secreting cells, was not different between lesions and NAWM, nor was the presence of plasma cell-associated CD138^+^ cells. Presence of NK cells could not be quantified due to their very limited presence in perivascular cuffs (Supplementary Fig. 2c, Supplementary Material 1). Overall, in addition to the abundance of CD4^+^ T cells in all perivascular cuffs, we see an increase of B-cell lineage cells in cuffs spatially associated with demyelinated lesions.

### Lymphocytes in perivascular cuffs show inflammatory activity

**Fig. 4:**
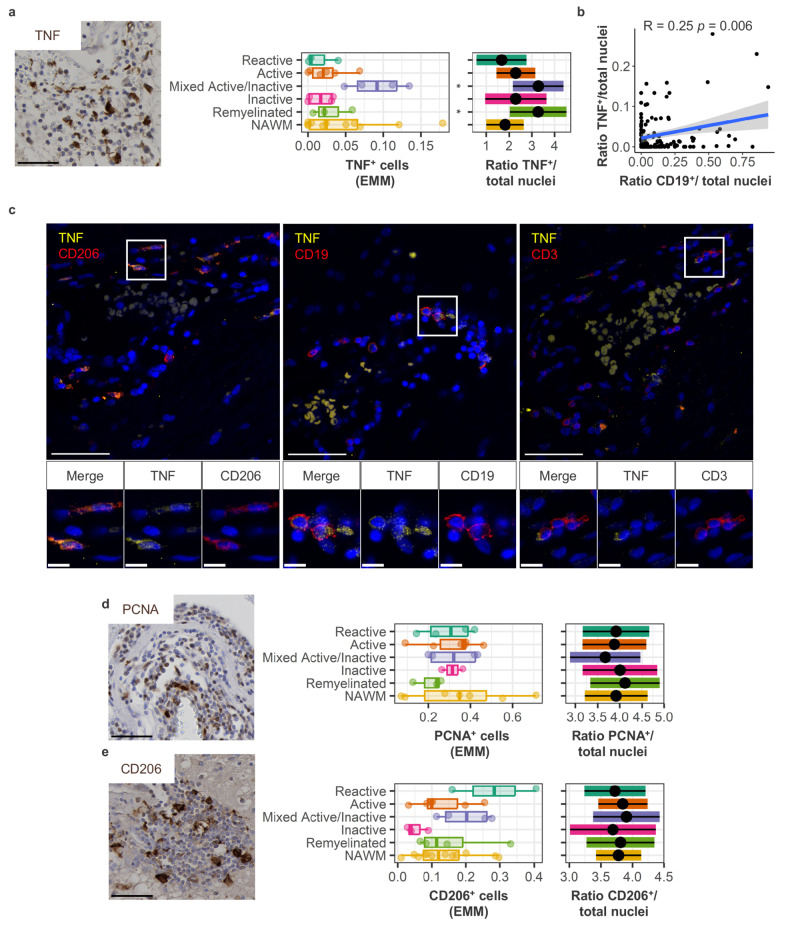
Increased TNF expression in mixed active/inactive lesions. **a** Representative staining and quantification of TNF^+^ cells in perivascular cuffs per WM lesion type compared to NAWM, **b** including a correlation of TNF^+^ cells with CD19^+^ cells. **c** Double staining of TNF with CD206^+^ perivascular macrophages, together with sporadic instances of TNF^+^ CD19^+^ B cells and TNF^+^ CD3^+^ T cells. **d** Representative staining and quantification of proliferation marker PCNA per WM lesion type compared to NAWM. **e** Representative staining and quantification of CD206^+^ perivascular macrophages per WM lesion type compared to NAWM. Scale bars indicate 50 μm, or 10 μm for magnified images. Plots are visualized as individual data point in a scatter plot, as average ratio of positive cells/counted nuclei per donor and estimated marginal means of absolute positive cell counts, a predicted adjusted mean within a model that takes into account nested data and variance in perivascular cuff size. Box plots show median and interquartile range. Statistics were performed using a linear mixed model using a negative binomial distribution or a Pearson correlation. Multiple testing correction was performed using a Benjamini-Hochberg FDR. * *p* < 0.05; EMM, estimated marginal means; NAWM, normal appearing white matter

To explore inflammatory activity in perivascular cuffs, we stained TNF for inflammatory cytokine production and PCNA for proliferation. TNF^+^ cells were enriched in perivascular cuffs within mixed active/inactive lesions (*p* = 0.017; Fig. 4a), which correlated with an increased presence of CD19^+^ cells in adjacent sections (*R* = 0.25; *p* = 0.006; Fig. 4b). Indeed, we sporadically found TNF^+^ B cells, as well as TNF^+^ T cells, in perivascular cuffs (Fig. 4c). Yet, the most common TNF^+^ cells are CD206^+^ macrophages (Fig. 4c). A substantial fraction of cells in perivascular cuffs were PCNA^+^, indicating ongoing proliferation, although this did not associate with presence of any lesion type (Fig. 4d). The potential to present antigen could not be quantified with HLA-DR stainings due to the ubiquitous presence of HLA within every perivascular cuff investigated (Supplementary Fig. 2c, Supplementary Material 1). To still assess antigen presentation potential, we quantified perivascular macrophages as professional antigen-presenting cells within the perivascular niche by staining for the mannose receptor CD206. No differences in CD206^+^ cell abundance between lesion types were noted. By observation, perivascular macrophages were scattered between lymphocytes and frequently located near the parenchymal edge of the perivascular space (Fig. 4e). We conclude that perivascular TNF production was associated with mixed active/inactive lesion activity, while proliferation and antigen-presentation associated markers were prominent within all perivascular cuffs.

### B cells in perivascular cuffs interact with T cells and produce IgG

**Fig. 5:**
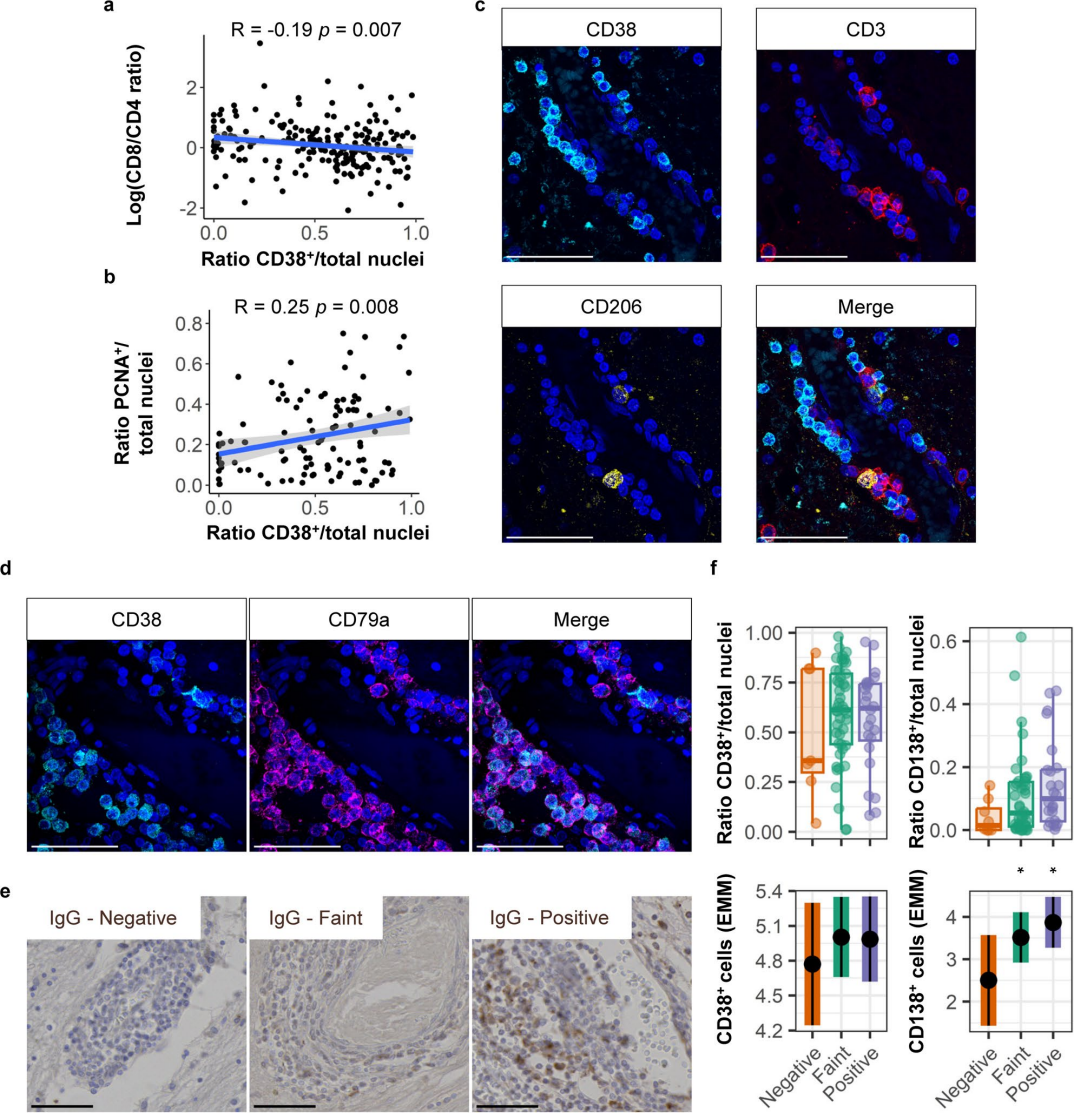
Intra-cuff correlations show T-cell-dependent activity state. **a** Correlation of CD8^+^/CD4^+^ T-cell ratio with CD38^+^ cell presence. **b** Correlation of CD38^+^ cells with the presence of proliferative PCNA^+^ cells. **c** CD38 double staining with CD3^+^ T cells or CD206^+^ perivascular macrophages, and **d** CD79a^+^ B cells. **e** Semi-quantification of immunoglobulin G in perivascular cuffs. **f** CD38^+^ and CD138^+^ antibody secreting cell abundance in relation to the presence of IgG in perivascular cuffs. Cuffs with IgG expression are compared to IgG negative cuffs. Scale bars indicate 50 μm. Plots are visualized as individual data point in a scatter plot, as average ratio of positive cells/counted nuclei per donor and estimated marginal means of absolute positive cell counts, a predicted adjusted mean within a model that takes into account nested data and variance in perivascular cuff size. Box plots show median and interquartile range. Statistics were performed using a linear mixed model using a negative binomial distribution or a Pearson correlation. Multiple testing correction was performed using a Benjamini-Hochberg FDR. *p < 0.05; EMM, estimated marginal means; IgG, immunoglobulin G

To explore interactions between lymphocytes, we correlated cell populations in sequential tissue sections. CD8^+^/CD4^+^ T-cell ratio was weakly correlated with the density of CD19^+^ cells (*R*= − 0.13, *p* = 0.041; Supplementary Fig. 2d, Supplementary Material 1) and CD38^+^ cells (*R*= − 0.19, *p* = 0.007; Fig. 5a). Moreover, CD38^+^ cell presence correlated positively with presence of PCNA^+^ cells (*R* = 0.25, *p* = 0.008; Fig. 5b). Since CD38 can be expressed by myeloid cells, antibody-secreting cells, and activated T cells, we performed double staining in three perivascular cuffs, showing that on average 85% (range: 81–93%) of CD38^+^ cells were also CD79a^+^ (Fig. 5c). Furthermore, in three perivascular cuffs, only two out of 100 CD38^+^ cells were CD3^+^, and no CD38^+^ cells co-expressed CD206 (Fig. 5d). Since CD79a and CD38 together mostly characterize B-lineage populations with an antibody-secreting phenotype, we stained for IgG (Fig. 5e). IgG presence in perivascular cuffs associated with increased density of CD138^+^ cells compared to IgG^−^ perivascular cuffs (positive: *p* = 0.018; faint: *p* = 0.036) but not with presence of CD38 (positive: *p* = 0.390; faint: *p* = 0.390; Fig. 5f). We conclude that a relatively higher CD4^+^ T-cell density correlated with higher numbers of B-cells, and activated and antibody secreting CD38^+^ cells, supporting the notion that B cells get T cell help to mature locally within perivascular cuffs.

### Occasional latent EBV infection of B cells in perivascular cuffs

**Fig. 6:**
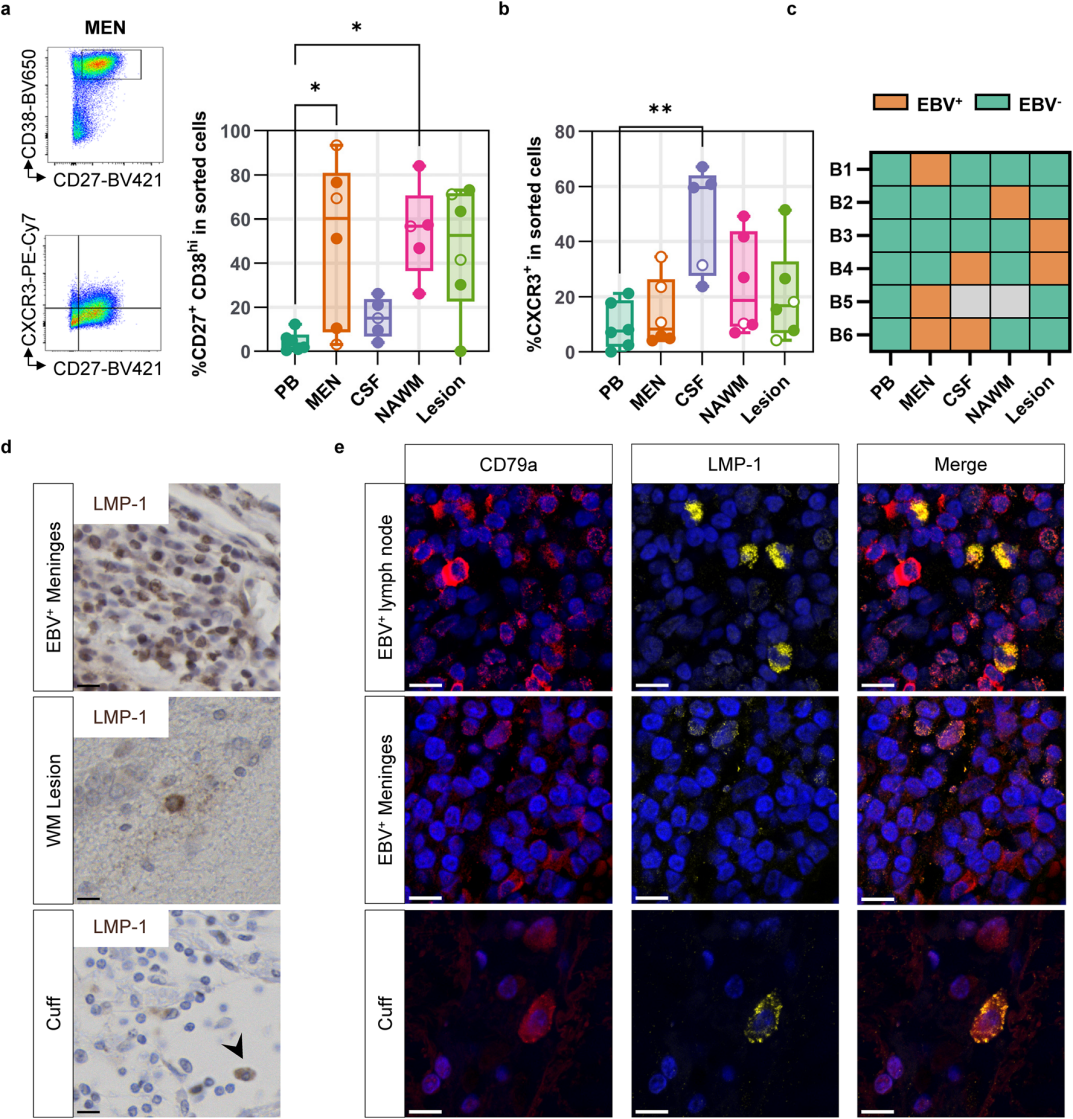
EBV presence is limited in perivascular cuffs. **a** CD38 and **b** CXCR3 positivity of isolated B cells. Shown are dot plots of the meningeal-derived CD19^+^/CD20^+^-cell fraction and positive percentages of all sorted cells. Data are from all sorted cells, including CD19^+^, CD20^+^, and CD19^−^CD20^−^CD38^hi^CD27^+^ cells. Open data points are EBV^+^. Statistical analysis was performed using a Kruskal-Wallis test with post hoc Dunn’s test. **c** Heatmap of EBV^+^ qPCR of isolated B cells from different CNS compartments of MS donors. **d** Stainings of EBV latent protein LMP-1. **e** Double staining of LMP-1 in CD79a^+^ B cells. * *p* < 0.05, ** p < 0.01; EBV, Epstein-Barr virus; WM, white matter. Scale bars indicate 10 μm

The recruitment of auto-proliferating Epstein-Barr virus (EBV)-infected B cells could drive the observed accumulation of perivascular B cells [22]. To investigate the presence of EBV in the CNS, we sorted viable B cells from fresh paired blood samples, meninges, CSF, NAWM, and WM lesions of MS donors and analyzed presence of viral genetic material using a multiplex real-time quantitative (q)PCR assay. Although CXCR3 is partially cleaved upon enzymatic digestion, these tissue compartments contained CD19^+^CD20^+^ B cells with a dominant CXCR3^+^ and CD38^hi^ phenotype as previously described for CNS-resident B cells, indicating we have primarily isolated CNS-associated B cells (Fig. 6a, b and Supplementary Fig. 2e, Supplementary Material 1) [9]. In contrast to blood, we detected EBV in B cells from at least one CNS compartment of all the 6 MS donors analyzed: 3 out of 6 meninges, 1 out of 5 CSF, 1 out of 5 NAWM, and 2 out of 6 WM lesion samples (Fig. 6c and Supplementary Table 3, Supplementary Material 1). To consolidate these findings, we investigated immunostaining of proteins expressed by EBV-infected B cells in different stages of infection [23]. As such, we stained tissue for the EBV latency proteins LMP-1 and EBNA2 in meninges of the earlier identified EBV^+^ donors of whom we earlier isolated viable B cells post-mortem. Subsequently, we stained these markers in the WM of donors with perivascular cuffs, of whom the stored tissue precluded isolation of viable B cells. We detected LMP-1^+^ cells in both tissues (Fig. 6d). Staining of LMP-1 did co-localize with CD79a confirming the B-cell identity of the LMP-1^+^ cells (Fig. 6e). Such clear positive staining was not present in meningeal tissue of a donor where we did not detect EBV using qPCR (Supplementary Fig. 2f, Supplementary Material 1). In perivascular cuffs, LMP-1^+^ B cells were found in 6 out of 11 donors analyzed (54.5%) and 13 out of 122 (10.7%) out of all perivascular cuffs investigated. This consisted mostly of a single positive cell and was found in all but remyelinated lesion types. EBNA2 was not detected outside of the positive control tissue (Supplementary Fig. 2g, Supplementary Material 1). These data confirm the presence of EBV^+^ B cells in perivascular cuffs of people with MS. However, as only a small fraction of perivascular B cells was LMP-1^+^, expansion of EBV-infected B cells is likely not the main driver of B-cell accumulation in cuffs.

### BTK expression correlates with CD19^+^ B-cell abundance in perivascular cuffs

**Fig. 7:**
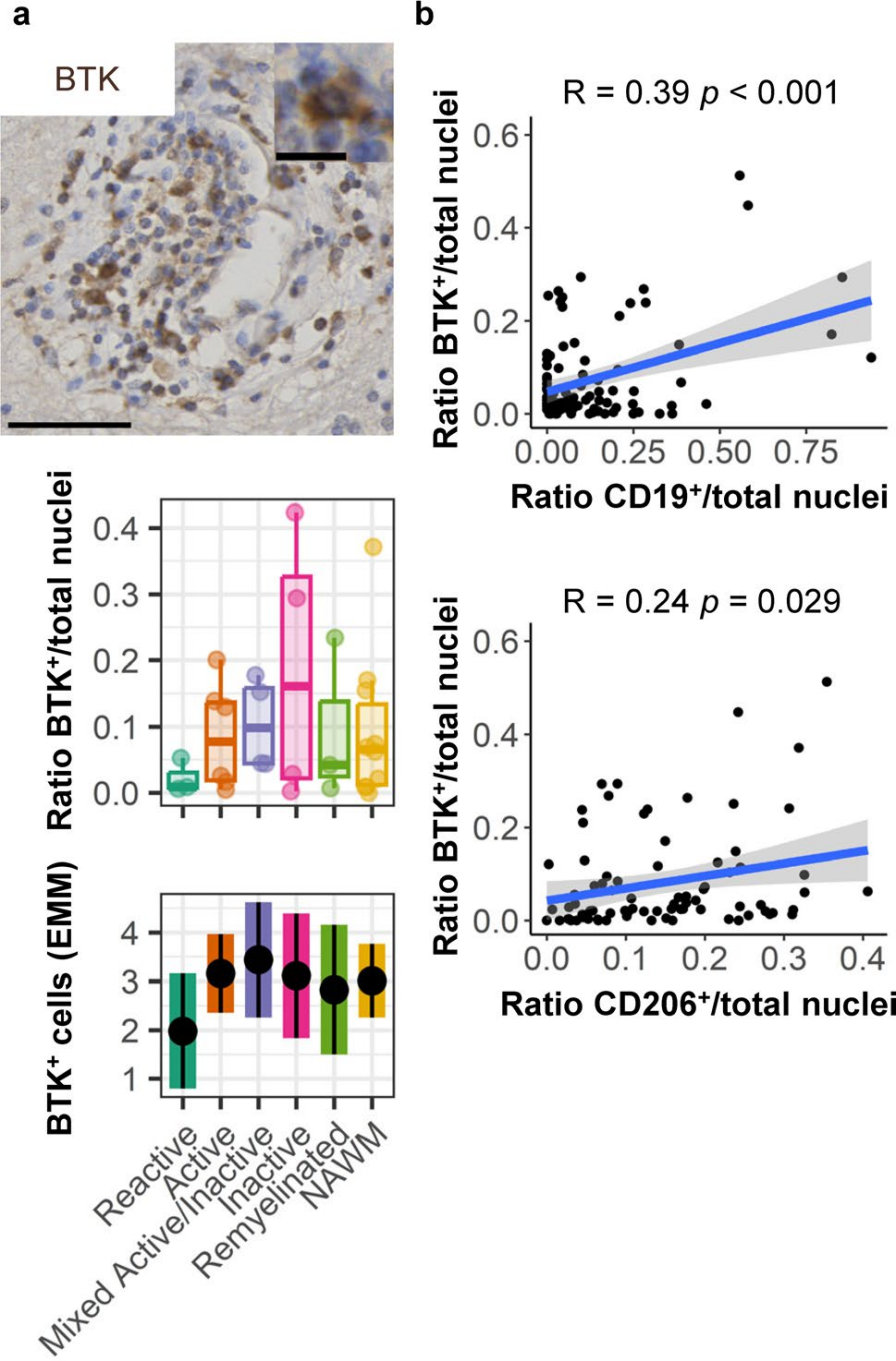
BTK presence in perivascular cuffs in relation to CD19^+^ and CD206^+^ cells. **a** Representative stainings and quantification of BTK^+^ cells per white matter lesion type compared to NAWM. **b** Correlation of BTK^+^ cell presence with the presence of CD19^+^ B cells and CD206^+^ perivascular macrophages. Scale bars indicate 50 μm–10 μm (magnification). Plots are visualized as individual data point in a scatter plot, as average ratio of positive cells/counted nuclei per donor and estimated marginal means of absolute positive cell counts, a predicted adjusted mean within a model that takes into account nested data and variance in perivascular cuff size. Box plot shows median and interquartile range. Statistics were performed using a linear mixed model using a negative binomial distribution or Pearson correlation. Multiple testing correction was performed using a Benjamini–Hochberg FDR. BTK, Bruton’s tyrosine kinase; EMM, estimated marginal means; NAWM, normal-appearing white matter

In addition to EBV, another molecule-of-interest in relation to the observed B cell presence in demyelinating lesions is Bruton’s tyrosine kinase (BTK). BTK is an important mediator of B-cell differentiation and survival [24], and treatment with brain-penetrating BTK-inhibitors has been shown to reduce disability accumulation in progressive MS [25]. Thus, we investigated the presence of BTK in perivascular cuffs to explore whether these BTK inhibitors might be able to target enclosed perivascular inflammation. Although BTK abundance was not different between lesion types (Fig. 7a), BTK correlated with abundance of CD19^+^ (*R* = 0.39, *p* < 0.001; Fig. 7b) and CD206^+^ cells (*R* = 0.24, *p* = 0.028; Fig. 7b), but not with presence of CD38^+^ (*R* = -0.15, *p* = 0.157) or CD138^+^ cells (*R* = 0.00, *p* = 0.984; Data not shown). A higher proportion of CD19^+^ cells relative to the total CD79a^+^ B-cell fraction correlated with a higher abundance of BTK (*R* = 0.30, *p* = 0.010; Supplementary Fig. 2d, Supplementary Material 1). BTK was expressed by both CD19^+^ B cells and CD206^+^ perivascular macrophages (Supplementary Fig. 2h, Supplementary Material 1). Overall, we confirm the presence of BTK in B cells and perivascular macrophages within perivascular cuffs which is supportive of a potential effect of these brain-penetrant compounds on events as taking place in MS perivascular cuffs.

## Discussion

We here provide a comprehensive characterization of perivascular cuffs in the WM of people with MS and shed light on the relevance of cuff composition and its spatial association with ongoing compartmentalized inflammation in advanced MS. MS donors with perivascular cuffs (25.5%) had a clinically and pathologically more severe disease indicated by a younger age at death, an increased presence of microglia nodules, and a higher brainstem lesion count. Compared to normal-sized PVS, the relative CD4^+^/CD8^+^ T-cell ratio in cuffs was increased irrespective of spatial association with lesions. B-lineage cells were abundant in perivascular cuffs and associated numerically with active demyelinated lesions. All perivascular cuffs showed correlates of cell proliferation and antigen presentation, and a high density of TNF^+^ cells characterized perivascular cuffs near mixed active/inactive lesions. A relative raise in CD4^+^/CD8^+^ T-cell ratio associated with a higher density of B cells and CD38-expressing antibody secreting cells. Although CD38 associated with more proliferating (activated) cells, an association with IgG production could not be shown. Perivascular-cuff-resident B cells sparsely expressed LMP-1 suggesting that EBV is unlikely to be the central driver of their proliferation in cuffs. Collectively, these data point to interaction between B and T cells in perivascular cuffs, which corroborates their proposed role as a hub for MS-lesion formation and maintenance of ongoing compartmentalized disease activity in advanced MS. The abundant expression of BTK in perivascular cuffs gives an exciting prospect for novel therapies to potentially modify disease-associated mechanisms in these cuffs.

The association of perivascular cuffs with more severe clinical characteristics and the correlation with more active lesions are in line with the previously described association of increased perivascular inflammation with disease severity [5, 7, 26, 27]. For instance, a previous report associated the presence of perivascular cuffs with more demyelination and an immune-dominant phenotype [13]. Overall, perivascular cuffs associate with a more severe disease course and an increase in tissue damage. In tandem, the co-occurrence of nodules with perivascular cuffs shows the increased propensity for lesion formation in donors with perivascular cuffs [21].

We report that CD4^+^ and CD8^+^ T-cell presence in perivascular cuffs is consistently high, ranging from NAWM to active demyelinating and inactive WM lesions. The increased abundance of CD4^+^ T cells suggests an important role in the formation of perivascular cuffs. This is in line with previous findings, where T cells in the PVS show an increase in transcriptomic signatures of T-cell activation and maintenance of T helper (T_h_) cells [28]. The prominent genetic susceptibility risk factor HLA-DRB1*15:01 and the implication of various CD4^+^ T-cell subsets underscore the relevance of CD4^+^ T cells for MS [29]. For instance, T_h_17.1 cells are associated with early MS and can readily cross the blood–brain barrier [30–32]. Additionally, follicular helper T cells have been identified in the brain of people with progressive MS [9, 33], and the CD4^+^ T_RM_ cells we described in MS brain tissue hold large phenotypic similarity with the recently identified T-peripheral helper cell, characterized by CCR2, CCR5, and PD-1 expression [17, 18]. Importantly, the relative presence of B cells (indicated by expression of CD19, CD20, and CD79a) was increased specifically in association with active demyelinated lesions compared to NAWM. This is concordant with the substantial suppression of lesion formation and progression throughout the course of MS in people treated with anti-CD20 monoclonal antibodies [34, 35]. B-cell activity in the PVS has indeed been associated with lymphocyte activation, immunoglobulin production, and somatic hypermutation using spatial sequencing [28]. In this way, the increased presence of perivascular B cells might reflect a WM homolog of the meningeal B-cell follicle [36, 37]. The increased number of CD79a^+^ B cells in inactive lesions compared to NAWM might be a remnant of previous B-cell contribution to lesions onset and activity. The presence of activation-associated CD38, widely expressed by memory B cells, is high overall and could represent differentiating antibody-secreting cells. This is in line with the abundance of CD27^+^CD38^hi^CD138^−^ antibody secreting cells found using flow cytometry [9]. Local maturation and persistence of antibody-secreting cells are distinctive features of the MS brain already present in NAWM [9]. In this study, we reiterate the positive correlation of CD4^+^ T cells to the presence of antibody-secreting cells in situ. Previous spatial sequencing analysis has revealed the presence of IgG^+^ CD79a^+^ B cells associated with foamy microglia, indicating that antibody secreting cells contribute to sustained neuroinflammation in MS [28]. In line, IgG isolated from myelin of post-mortem brain tissue was able to break tolerance of microglia to stimulation with LPS or poly(I:C) [38]. Yet, we do not show an association of IgG presence with CD38, potentially due to the difficult nature of IgG stainings or a population of CD38^+^ B cells, which have not yet undergone class switching. Potentially, the unique nature of perivascular cuffs allows for this due to more local immune interaction and activation.

Together, our data indicate that T–B-cell interaction in the PVS happens locally in perivascular cuffs independent of lesion formation. Yet, this can still be an important disease mechanism in MS, as seen by increased CD19 abundance in mixed active/inactive lesions. Namely, T–B-cell interaction can lead to activation and proliferation of T cells [32], and can be seen in the PVS of people with MS in situ through CXCR5 expression [9]. This is also reflected by reports that CXCR3^+^ B cells in MS can interact with T cells, form clusters, and develop into antibody-secreting cells *in vitro* [10]. These atypical B cells have also been found to have a high potential for cytokine production [39]. Although not linked to direct production by B cells, this is reflected by the increased expression of TNF in mixed active/inactive lesions. Although TNF is also increased in remyelinated lesions, we do not further investigate TNF there due to the low effect size and low number of cuffs in remyelinated lesions. Within cuffs, TNF is majorly expressed by macrophages, although there are sporadic TNF^+^ B- and T cells present within perivascular cuffs. The correlation of CD19^+^ B cells with presence of TNF does not imply the direct production of cytokines by local B cells, but rather highlights the pro-inflammatory nature of perivascular cuffs in mixed active/inactive lesions. The local production of soluble factors by perivascular cells has been proposed as main driver of MS-lesion activity [40], where TNF and B-cell exosomes have been hypothesized as candidates [41, 42]. This leads us to hypothesize that perivascular cuffs form in the MS brain because of local T–B-cell interaction and can be associated with antigen presentation, cytokine production and parallel local B-cell activation, proliferation, and differentiation. As a result, local antibody production within the central nervous system could drive progression through breaking myeloid cell immune tolerance [38]. Together, this would create a vicious cycle of damage and immune activation.

Previous reports indicate abundant presence of EBV^+^ cells in perivascular cuffs and ectopic lymphoid follicles [22]. Yet, these results were not replicated within the same cohort [43]. This confirms the challenging nature of detecting EBV presence in the CNS. More recently, another paper showing the presence of EBV in MS lesions emerged, stating an enrichment of LMP-1 and EBNA1 within MS lesions, including co-staining with B cells, reactive astrocytes, microglia and neurons [44, 45]. Here we confirm the occasional presence of EBV^+^ B cells within the CNS of a subgroup people with MS using multiplex real-time qPCR on isolated B cells with high CXCR3 expression. By staining meninges of EBV^+^ donors, we provide indirect evidence of LMP-1 being a biomarker for detection of EBV^+^ B cells in the CNS. We did not perform EBER stainings [22], due to technological challenges as risk of false positive findings [43]. EBNA2^+^ B cells were not detected within our CNS tissue, which could be due to a skewed distribution of latency stages. Further exploration of different EBV-latency stages in CNS/meningeal B cells, including stage 0 and 1 as reflected by EBNA1, remains to be studied in further work [46]. The earlier published BZLF1-antibody did not work in our hands [45]. Yet, when studying perivascular cuffs LMP-1^+^ cells were infrequently found, suggesting that a massive accumulation and proliferation of EBV-infected B cells is an unlikely explanation of perivascular cuff formation in MS.

Although BTK is not increased in perivascular cuffs near demyelinating lesions, it is correlated with the presence of CD19^+^ cells. One explanation could be that not BTK expression per se but rather phosphorylation of BTK, as is associated with BTK activity, and B-cell differentiation associate with lesion formation in MS [47]. In light of trials with BTK inhibitors that show an ameliorating effect on disease progression [25, 48], a proposed mechanism of brain-infiltrating BTK inhibitors could be the prevention of cluster formation and proliferation or antibody secreting cell formation in situ. This is reinforced by reports that the formation of T–B-cell clusters can be prevented *in* vitro by BTK-inhibitor ibrutinib [10]. Since BTK is also expressed in microglia, where BTK inhibitors can block inflammatory signaling [49], another interest is BTK expression in perivascular macrophages. These could interact with the local B-cell population and potentiate CNS inflammation [50]. Thus, selective BTK inhibitors have the potential to act upon compartmentalized inflammation not only through the B-cell lineage, but also through myeloid cells. Overall, our results add to the exciting prospect of BTK inhibition to target enclosed PVS inflammation in progressive MS.

There are some limitations to this study. The sample size is limited in number of brain donors, yet analyzed a substantial number of perivascular cuffs. Throughout sections, some perivascular cuffs might be lost, acquired, or merged. This is mitigated by our statistical approach. Additionally, the donor cohort comprises donors with advanced MS, providing an excellent cohort to analyze advanced, compartmentalized inflammation. Moreover, we did not include B cell and LMP-1 quantifications of non-MS donors since the presence of B cells in control white matter is a very rare event [8]. Furthermore, there is a possibility of false positive LMP-1 staining due to the high abundance of IgG in the cytoplasm of plasma blasts/cells. We mitigated this limitation by confirming presence of EBV-positive B cells in CNS compartments with qPCRs. BTK phosphorylation was not quantified. Last, substantial variation between perivascular cuffs and analysis of serial sections resulted in relatively weak correlations reported, mitigated by the large numbers of perivascular cuffs analyzed.

Altogether, our findings enforce the perivascular cuff as a relevant MS characteristic associating with a more severe disease course and ongoing lesion-activity in MS. The presence of perivascular cuffs is identified as a donor-trait for a more inflammatory active disease, with a local relative overabundance of CD4^+^ T cells. These T cells might interact with a local B-cell population, which is most pronounced in mixed active/inactive lesions, leading to activation, differentiation and proliferation. In sum, our study gives insight in the processes occurring in perivascular cuffs which are associated with local ongoing inflammation in MS. Perivascular B cells and macrophages residing in the brain can also express BTK and might contribute to the formation of perivascular cuffs, reaffirming the therapeutic potential of BTK inhibitors as reported in recent MS trials.

## Supplementary Information

Below is the link to the electronic supplementary material.

Supplementary Material 1.

## Data Availability

The data that support the findings of this study are available from the corresponding author upon reasonable request.

## Abbreviations

BTK: Bruton’s tyrosine kinase
CNS: Central nervous system
EBV: Epstein-Barr virus
EMM: Estimated marginal means
F: Female
gARMSS: Global age-related MS severity score
IgG: Immunoglobulin G
M: Male
mA/I: Mixed active/inactive lesions
MS: Multiple sclerosis
MMAS: Microglia/macrophage activation score
NAWM: Normal appearing white matter
NBB: Netherlands Brain Bank
PP: Primary progressive
PVS: Perivascular space
SP: Secondary progressive
T_RM_: Tissue-resident memory T cell
WM: White matter
