# Supplementary material for: B-cell abundance in perivascular cuffs associates with local lesion activity in multiple sclerosis
Source: Acta Neuropathol Commun. 2026 Feb 5;14:69. doi: 10.1186/s40478-025-02208-4 (PMC13019733; doi:10.1186/s40478-025-02208-4)
Supplement: Supplementary file 1 — Supplementary Material 1. [file 40478_2025_2208_MOESM1_ESM.pdf]

Supplementary Material 1: Suppl. Figures 1-2, Suppl. Tables 1-3

Article title: B-cell abundance in perivascular cuffs associates with local lesion activity in multiple sclerosis

Journal name: Acta Neuropathologica Communications

Author names: Hendrik J. Engelenburg, Esmée Westenbrink, Eline Runderkamp, Ana M. Marques, Marvin M. van Luijn, Cheng-Chih Hsiao, Inge Huitinga, Jörg Hamann, and Joost Smolders

Corresponding author: H.J. Engelenburg or J.J.F.M. Smolders, Neuroimmunology research group, Netherlands Institute for Neuroscience, Meibergdreef 47, 1105 BA Amsterdam, The Netherlands, j.engelenburg@nin.knaw.nl or j.j.f.m.smolders@erasmusumc.nl

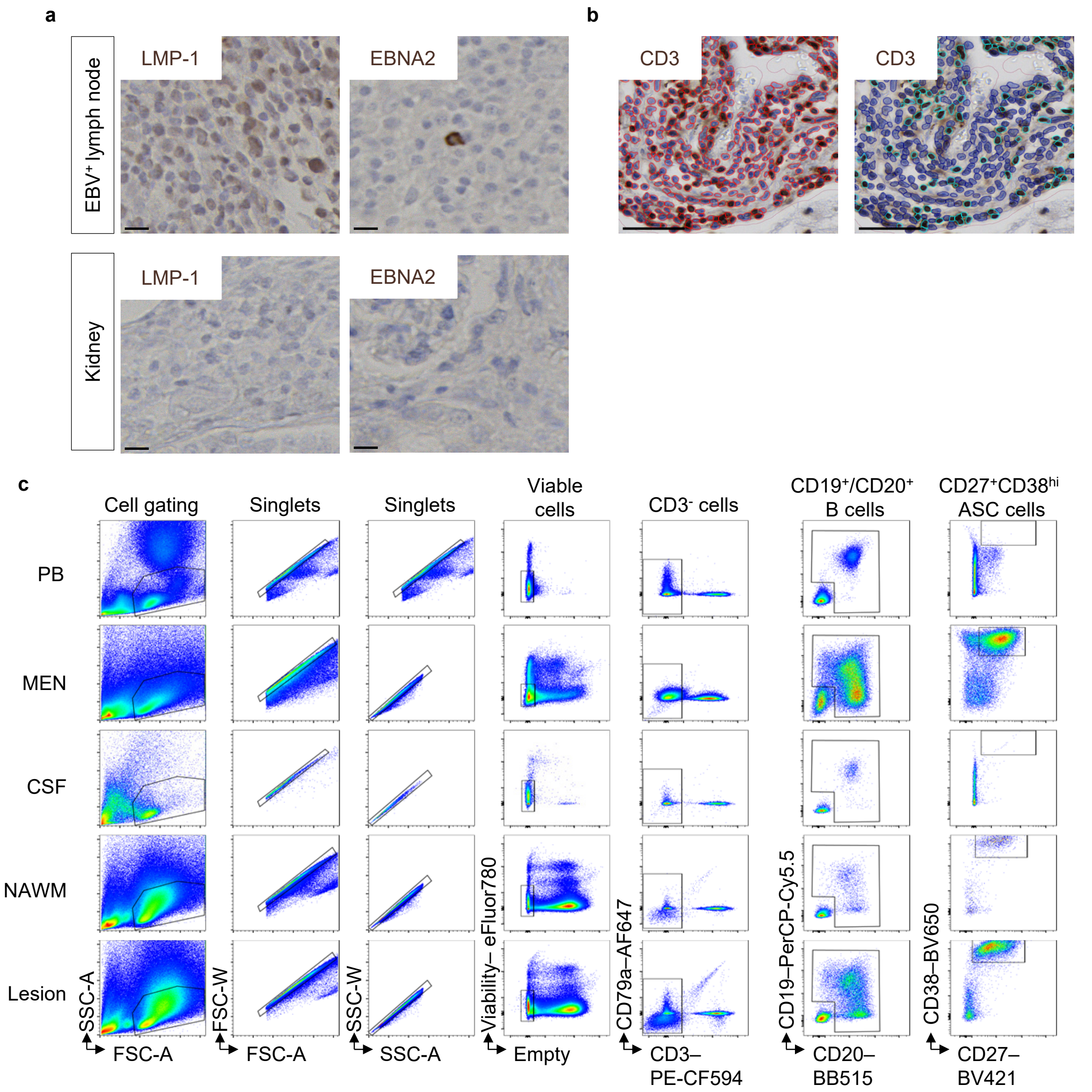

Supplementary Fig. 1 Method details. (**a**) Positive and negative control of EBV latent protein stainings. As positive control, a lymph node of someone with infectious mononucleosis was used and as negative control a kidney with no apparent EBV expression. Scale bars indicate 10 µm. (**b**) Image of nuclei detection and thresholding algorhitm on a CD3 staining. In red all detected nuclei are annotated, with the lower image showing in navy cells that were identified as CD3$^-$ and in cyan cells that were identified as CD3$^+$. Scalebars indicate 50 µm. (**c**) Representative dot plots showing the gating strategy used for flow cytometry with, from left to right, cell gating by forward scatter (FSC) and sideward scatter (SSC), FSC-area/width duplet exclusion, SSC-area/width duplet exclusion, gating of viable cells, gating of viable CD3$^-$ cells, gating of viable CD19$^+$/CD20$^+$ B cells, and gating of viable CD27$^+$CD38^{hi} antibody-secreting cells (ASC) cells. MEN, meninges; NAWM, normal-appearing white matter

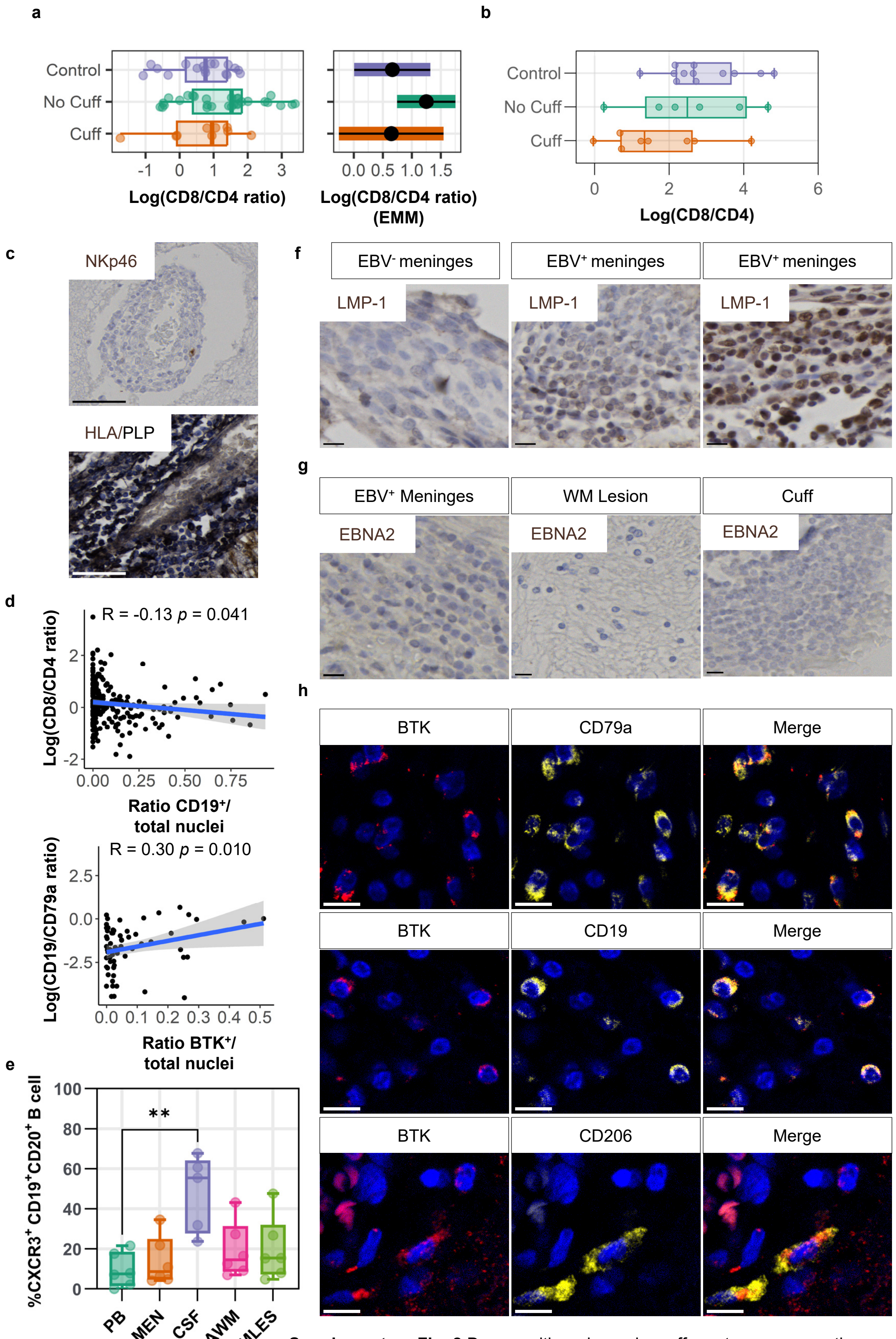

Supplementary Fig. 2 Donors with perivascular cuffs portray a more active pathology. (**a**) CD8/CD4 ratio in MS NAWM or control WM as determined by immunohistochemistry stratified between donors with and without perivascular cuffs. No significant difference was observed. Statistical test was a linear model with a Gaussian distribution. Data from: Fransen *et al.*, Brain 2020. (**b**) CD8/CD4 ratio as determined by flow cytometry stratified between control donors and MS donors with and without cuffs. No significant difference was observed. Statistical test consisted of Wilcoxon rank sum tests. Data from: Fransen *et al.*, Brain 2020.; Hsiao & Engelenburg *et al.,* iScience 2023; Pignata *et al.,* Sci Transl Med 2025. (**c**) Limited NK cell and abundant HLA presence in perivascular cuffs as seen with immunohistochemistry. (**d**) positive (Pearson) correlation of CD19 density compared to the relative abundance of $CD4^+$ T cells and of BTK density compared to relative abundance of $CD19^+$ B cells. (**e**) Higher CXCR3 expression in CNS-derived isolated $CD19^{+/-}CD20^{+/-}$ B cells. Statistics were performed using a Kruskal-Wallis test with post-hoc Dunn's test. (**f**) Additional stainings of Epstein-Barr virus latent protein LMP, showing an LMP-1$^-$ cluster in EBV$^-$ and EBV$^+$ meninges, and an additional LMP-1$^+$ cluster in EBV$^+$ meninges. (**g**) Stainings of Epstein-Barr virus latent protein EBNA2. (**h**) Double staining of BTK with B cell markers (CD79a, CD19) and perivascular macrophage marker (CD206) in a perivascular cuff shows its expression by both celltypes. Scale bars indicate 50 µm (DAB: NKp46 &HLA/PLP) or 10 µm (fluorescent & DAB: EBNA2). ** $p<0.01$. EMM, estimated marginal means.

Supplementary Table 1. Overview of brain donor cohort

	Sex	Age (Years)	PMD (hh:mm)	pH of CSF	Brain weight (g)	Diagnosis	Use
Perivascular cuff cohort	6M/12F	56.1 ± 12.7	08:10 ± 01:47	6.57 ± 0.21	1246 ± 117	2 N/A, 1 RR, 7 PP, 8 SP	IHC
B cell isolation cohort	1M/5F	61.5 ± 10.3	07:03 ± 01:25	6.46 ± 0.17	1231 ± 146	1 N/A, 1 PP,	RQ-PCR/ IHC

Values shown are mean ± standard deviation. CSF, cerebrospinal fluid; F, female; IHC, immunohistochemistry; M, male; N/A, not available; PP, primary progressive MS; RQ-PCR, real-time quantitative polymerase chain reaction; RR, Relapsing Remitting MS; SP, secondary progressive MS.

Supplementary Table 2. Overview of antibodies

Antigen	Supplier (cat#)	Host	Clone	Dilution	Antigen retrieval	Use
Anti-human BTK	Cell Signaling Technology (8547)	Rabbit	D3H5	1:100	Citraconic anhydride pH7.6	IHC
Anti-human CD3	Dako (A0452)	Rabbit	Polyclonal	1:100	Citrate pH6.0	IHC
Anti-human CD3	Abcam (Ab11089)	Rat	CD3-12	1:200	Citraconic anhydride pH7.6	IHC
Anti-human CD4	Abcam (ab133616)	Rabbit	EPR6855	1:500	Citrate pH6.0	IHC
Anti-human CD8	Abcam (ab4055)	Rabbit	Polyclonal	1:200	Citrate pH6.0	IHC
Anti-human CD19	Dako (M7296)	Mouse	LE-CD19	1:100	Citraconic anhydride pH7.6	IHC
Anti-human CD19	Abcam (ab134114)	Rabbit	EPR5906	1:200	Tris-EDTA pH 9.0	IHC
Anti-human CD20	Dako (M0755)	Mouse	L26	1:100	TBS pH7.6	IHC
Anti-human CD38	Atlas antibodies (HPA022132)	Rabbit	Polyclonal	1:2000	Citrate pH6.0	IHC
Anti-human CD79a	Dako (M705001-2)	Mouse	JCB117	1:200	Tris-EDTA pH9.0	IHC
Anti-human CD79a–Alexa Fluor 647	BioLegend (333515)	Mouse	HM47	1:200	Citrate pH6.0	IHC
Anti-human CD138	BioRad (MCA2459T)	Mouse	B-A38	1:500	Citrate pH 6.0	IHC
Anti-human CD206	Abnova (H00004360-M02)	Mouse	5C11	1:1600	Citraconic anhydride pH7.6	IHC
Anti-human CD206	Abcam (ab64693)	Rabbit	Polyclonal	1:1000	Tris-EDTA pH 9.0	IHC
Anti-human EBNA2	Abcam (ab90543)	Mouse	PE2	1:200	Citrate pH 6.0	IHC
Anti-human PCNA	Santa Cruz (sc-56)	Mouse	PC10	1:1000	Citrate pH 6.0	IHC
Anti-human HLA-DR,DP,DQ	Abcam (ab7856)	Mouse	CR3/43	1:100	TBS pH7.6	IHC
Anti-human IgG	Abcam (ab218427)	Rabbit	IG507R	1:200	Citraconic anhydride pH7.6	IHC
Anti-human LMP-1[#]	Abcam (ab78113)	Mouse	CS1-4	1:800	Citrate pH6.0	IHC
Anti-human NKp46	R&D systems (MAB1850)	Mouse	195314	1:100	Citrate pH6.0	IHC
Anti-human PLP	BioRad (MCA839G)	Mouse	PLPC1	1:3000	TBS pH7.6	IHC
Anti-human TNF	Proteintech (60291-1-Ig)	Mouse	7B8A11	1:500	Tris-EDTA pH 9.0	IHC

Biotinylated-anti mouse (rat absorbed)	Vector Laboratories (BA-2001)	Horse	Polyclonal	1:400	N/A	IHC
Streptavidin-Alexa Fluor 488	Jackson ImmunoResearch (016-540-084)	N/A	N/A	1:1200	N/A	IHC
Anti-rabbit-Cy3	Jackson ImmunoResearch (711-165-152)	Donkey	Polyclonal	1:800	N/A	IHC
Anti-rat-Cy5	Jackson ImmunoResearch (712-606-153)	Donkey	Polyclonal	1:800	N/A	IHC
Anti-rabbit-Alexa Fluor 647	Jackson ImmunoResearch (711-606-152)	Donkey	Polyclonal	1:800	N/A	IHC
Anti-mouse-Cy3	Jackson ImmunoResearch (715-166-150)	Donkey	Polyclonal	1:800	N/A	IHC
Anti-mouse Alexa Fluor 488	Jackson ImmunoResearch (715-546-150)	Donkey	Polyclonal	1:800	N/A	IHC
Anti-rabbit Alexa Fluor 594	Jackson ImmunoResearch (711-585-152)	Donkey	Polyclonal	1:800	N/A	IHC
LIVE/DEAD Fixable Viability Dye eFluor 780	Thermo Fisher Scientific (65-0865)	N/A	N/A	1:1000	N/A	Flow cytometry
Anti-human CD79a– AF647	BioRad (MCA1298A647T)	Mouse	ZL7-4	1:25	N/A	Flow cytometry
Anti-human CD3–PE-CF594	BD Biosciences (562280)	Mouse	UCHT1	1:500	N/A	Flow cytometry
Anti-human CD19–PerCP-Cy5.5	BioLegend (302229)	Mouse	HIB19	1:200	N/A	Flow cytometry
Anti-human CD20–BB515	BD Biosciences (564569)	Mouse	2H7	1:200	N/A	Flow cytometry
Anti-human CD38–BV650	BD Biosciences (569391)	Mouse	HIT2	1:100	N/A	Flow cytometry
Anti-human CD27–BV421	BioLegend (302823)	Mouse	O323	1:100	N/A	Flow cytometry
Anti-human CD45–R718	BD Biosciences (566962)	Mouse	HI30	1:100	N/A	Flow cytometry
Anti-human CD138–PE	BioLegend (356503)	Mouse	MI15	1:50	N/A	Flow cytometry
Anti-human CXCR3–PE-Cy7	BioLegend (353719)	Mouse	G025H7	1:100	N/A	Flow cytometry

Cat#, category number; IHC, immunohistochemistry; N/A, not available; TBS, tris-buffered saline; # indicates 30 minutes blocking with methanol/3% H_2O_2.

Supplementary Table 3. DNA concentration (ng) and EBV positivity of isolated B cells

Brain donor	PB		Meninges		CSF		NAWM		WM Lesion	
Donor B1	142	-	629	+	122	-	339	-	222	-
Donor B2	142	-	656	-	163	-	656	+	325	-
Donor B3	142	-	952	-	139	-	1374	-	755	+
Donor B4	178	-	838	-	103	+	130	-	117	+
Donor B5	257	-	341	+	N/A		N/A		386	-
Donor B6	236	-	2.044	+	166	-	408	-	689	-

CSF, cerebrospinal fluid; MEN, meninges; NAWM, normal appearing white matter; PB, peripheral blood; WM, white matter; +/– indicates EBV status of sample.
